# Trends in the Use of Lipases: A Systematic Review and Bibliometric Analysis

**DOI:** 10.3390/foods12163058

**Published:** 2023-08-15

**Authors:** Lucely Nogueira dos Santos, Rafael Firmani Perna, Ana Carolina Vieira, Alex Fernando de Almeida, Nelson Rosa Ferreira

**Affiliations:** 1 Postgraduate Program in Food Science and Technology, Institute of Technology, Federal University of Pará (UFPA), Belém 66075-110, Brazil; lucelynogueira@gmail.com; 2 Graduate Program in Chemical Engineering, Institute of Science and Technology, Federal University of Alfenas (UNIFAL-MG), Poços de Caldas 37715-400, Brazil; rafael.perna@unifal-mg.edu.br (R.F.P.); ana.vieira@unifal-mg.edu.br (A.C.V.); 3 Engineering of Bioprocesses and Biotechnology, Federal University of Tocantins (UFT-TO), Gurupi 77402-970, Brazil; alexfernando@mail.uft.edu.br; 4 Faculty of Food Engineering, Institute of Technology, Federal University of Pará (UFPA), Belém 66075-110, Brazil

**Keywords:** bibliometric study, lipase, immobilization, industrial applications, scaling up, VOSviewer

## Abstract

Scientific mapping using bibliometric data network analysis was applied to analyze research works related to lipases and their industrial applications, evaluating the current state of research, challenges, and opportunities in the use of these biocatalysts, based on the evaluation of a large number of publications on the topic, allowing a comprehensive systematic data analysis, which had not yet been conducted in relation to studies specifically covering lipases and their industrial applications. Thus, studies involving lipase enzymes published from 2018 to 2022 were accessed from the Web of Science database. The extracted records result in the analysis of terms of bibliographic compatibility among the articles, co-occurrence of keywords, and co-citation of journals using the VOSviewer algorithm in the construction of bibliometric maps. This systematic review analysis of 357 documents, including original and review articles, revealed studies inspired by lipase enzymes in the research period, showing that the development of research, together with different areas of knowledge, presents good results related to the applications of lipases, due to information synchronization. Furthermore, this review showed the main challenges in lipase applications regarding increased production and operational stability; establishing well-defined evaluation criteria, such as cultivation conditions, activity, biocatalyst stability, type of support and reactor; thermodynamic studies; reuse cycles; and it can assist in defining goals for the development of successful large-scale applications, showing several points for improvement of future studies on lipase enzymes.

## 1. Introduction

Enzymes can act as natural catalysts on specific or non-selective substrates, usually under mild temperature, pressure, and pH conditions, and achieve high conversion rates [1]. In this context, lipases (triacylglycerol ester hydrolases EC 3.1.1.3) are widely distributed and studied. According to the reaction medium, lipases develop different activities, such as hydrolytic activity (aqueous medium), synthesis in esterification reactions (aqueous-organic medium), or transesterification or interesterification activity (organic medium) [2]. Lipases can also catalyze the direct interaction of carboxylic acids and hydrogen peroxide with the formation of peroxycarboxylic acids [3,4]. They can also transfer the acyl groups of complex esters by other nucleophiles, such as amines and thiols [5,6].

Lipases demonstrate several interesting properties, such as their ability to act at the interface between aqueous and non-aqueous phases, their ability to process all types of glycerides and free fatty acids in transesterification, specificity, high catalytic activity under milder reaction conditions, stability in organic solvents, and activity without cofactors. These properties of lipases demonstrate their good performance when applied in biocatalysis in the most diverse processes [4]. For example, in studies on biolubricants [7], the synthesis of glycerin monostearate [8], biosurfactant production [9], in the pretreatment of industrial [10], in the production of new nanobiocatalysts [11], and in the modification of cheese by enzymes [12].

Due to the diversity of catalytic processes involving lipases, a large number of studies have been published proposing improvements in the characteristics and mechanisms of action of these biocatalysts, such as improved stability [13,14], immobilization systems [15,16,17], and their applications in various biochemical processes for the food, pharmaceutical, leather, cosmetics, detergents, medical diagnostics, dairy, beverages, fatty acids, and paper industries [5,18,19,20]. Nonetheless, despite the large number of published studies and the critical contributions of lipases and their applications, there is still a tiny number of publications related to scientific mapping which aim to analyze the relationships among the constituents of the research. For example, research networks that involve keywords, the relationship between two articles based on the number of references in common, and the co-citation map of journals show the structure of the scientific areas researched. In this study, it was possible to map, organize, and extract information from large numbers of documents, clearly showing information and connections among the researched niches, with the support given by the bibliometric analysis tool. 

Bibliometric analysis is a quantitative method that uses mathematical and statistical tools to measure the interrelationships and impacts of publications within a given area of research, resulting in a macroscopic view of a large number of academic works. VOSviewer is a program designed to build and visualize bibliometric maps based on network data, considering the strength of the connection between items [21].

The emergence of scientific databases, such as Scopus and Web of Science, made it relatively easy to acquire large volumes of bibliometric data. This phenomenon associated with bibliometric software enables extensive data analysis, motivating academic interest in bibliometric analysis [22]. Therefore, this type of analysis has been applied based on keywords [12] and the citation index [23].

The present study highlights the relationship of study networks involving lipase enzymes, based on a search of the literature in English in the Web of Science (WS) database from 2018 to 2022 to highlight, through scientific mapping, the most researched topics involving lipases. With this, it was possible to identify the challenges related to the scale-up of the process catalyzed by lipases and to show perspectives for future research that seek to increase the production and operational stability of processes enzymatic.

## 2. Research Process and Scientific Mapping

Bibliometric analysis is a quantitative method that uses mathematical and statistical tools to measure the interrelationships and impacts of publications within a given area of research, resulting in a macroscopic view of a large number of academic literature works [21].

The data collected for this research was based on WS. The search expressions form the terms “lipase” and “industrial applications” for the topics in this field, which performs word searches in titles, abstracts, and keywords. According to Gonçalves et al. [24,25,26], the search for terms in quotation marks associated with Boolean operators allows searching for publications containing the words in an associated form and the exact search order.

**Figure 1:**
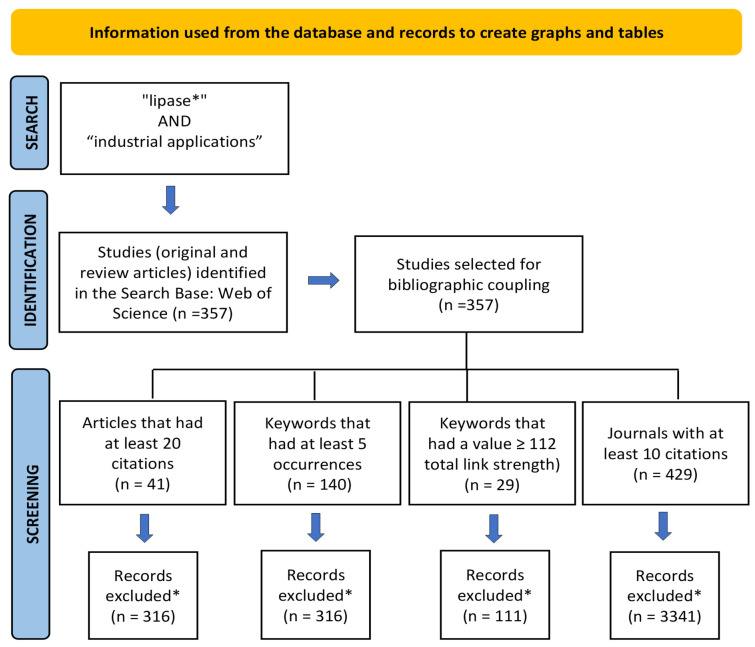
PRISMA flow diagram information used from the database and records to create charts and tables. * Records deleted by the VOSviewer program.

According to Uman [27], systematic reviews are characterized as studies and point directions for future investigations and the development of research projects in a particular area of study, using systematized strategies of search, analysis, and synthesis of literature data on a topic. The search was refined using the “type of documents” filter of WS, where “articles and review articles” were chosen. As a result, 357 publications were retrieved between 2018 and 2022. The records were exported and analyzed by the VOSviewer program version 1.6.17. In order to analyze the relationships between the research constituents, scientific mapping was performed using the analysis of bibliographic coupling between articles, co-occurrence of keywords, and co-citation of journals. The criteria for creating graphs and tables using VOSviewer are described in the PRISMA flow diagram [28] (Figure 1).

## 3. Bibliographic Coupling between Articles

**Figure 2:**
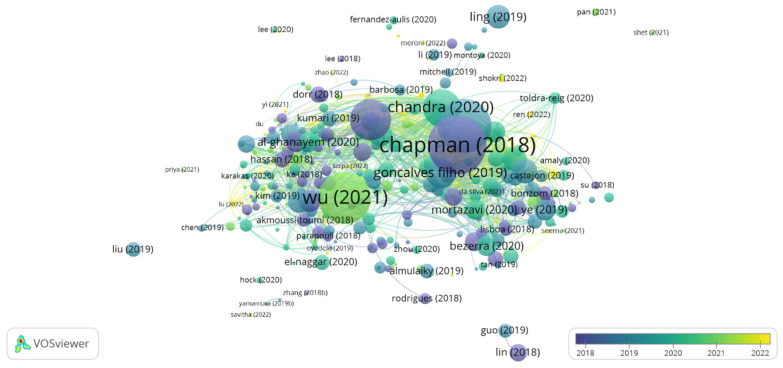
Bibliographic coupling: overlay view for articles from the period 2018 to 2022. The items’ size indicates the number of citations of the articles [1,5,13,30,31,32,33,34,35,36,37,38,39,40,41] Thus, the more citations an item had, the larger its label and circle.

Bibliographic coupling measures the proximity between two articles by comparing their references. Therefore, the greater the number of references they share, the greater the similarity between them, which can be theoretical, methodological, or another shared particularity [29]. Figure 2 shows the network visualization map considering 357 documents extracted from the WS.

The articles are indicated by a label and, by default, a circle. Thus, the more citations the document has, the larger the circle in the bibliographic coupling will be. The articles by the authors Chapman et al. [30], Basso and Serban [42], Wu et al., [36], Sarmah et al., [32], and Chandra et al. [5] were the most cited in the research period. These are review articles that discuss the advantages of enzyme immobilization and the challenges of implementing it in large-scale processes [30,42], as well as the main biocatalytic applications on an industrial scale [13] and structure, classification, and production [5,32]. Therefore, it was possible to perceive a wide spectrum of topics involving lipases in studies focused mainly on enzyme immobilization techniques and the potential applicability of these enzymes in various industrial processes.

In the bibliographic coupling chart, the strength of a link indicates the number of cited references that two publications have in common [43]. Table 1 presents the information of the articles that had at least 20 citations and their respective total values of binding strength based on the information presented in the articles by the authors [13,32,38]. These documents presented a value ≥ 700 for the total link strength, evidencing a connection network established among these studies. The total link strength value is not related to the number of citations of the article, but rather to the network of citations between two documents when they refer to at least one publication in common. Articles that presented values ≥ 700 for total link strength are mainly review papers in which the authors carefully analyzed the literature [5].

Chandra et al. [5] and Mahfoudhi et al. [44] reported that microorganisms efficiently produce lipolytic enzymes. On the other hand, increasing applications of lipases in biotechnology have been observed, such as in the hydrolysis of global residues, mainly plastic residues that disintegrate into microplastics and cause damage to flora and fauna [45]. Studies on the trends and advances in the field of enzymes and research with immobilized lipases and their effects on optimizing enzyme biocatalysts were reported by Sarmah et al. [32]. Techniques used for the immobilization of lipases, advances in research on support materials, and applications of these biocatalysts were described by Facin et al. [38]. The original article by Mortazavi and Aghaei [13] on the development of suitable surfaces for immobilizing α-amylase and lipase via the technique of adsorption presented a high total link strength value. 

In this context, from the connection network established among the articles, it is possible to observe the similarity of the research topics developed by these authors, evidenced by the total link strength value shown in bibliographic coupling.

**Table 1:** Articles with at least 20 citations and their respective total link strength values.

Article Title	Journal ISO Abbreviation	Citations	Total Link Strength	Authors
Industrial Applications of Enzymes: Recent Advances, Techniques, and Outlooks	*Catalysts*	300	359	[30]
Industrial applications of immobilized enzymes-A review	*Mol. Catal.*	256	137	[42]
Biocatalysis: Enzymatic Synthesis for Industrial Applications	*Angew. Chem. Int. Edit.*	240	265	[36]
Recent advances on sources and industrial applications of lipases	*Biotechnol. Prog.*	149	733	[32]
Microbial lipases and their industrial applications: a comprehensive review	*Microb. Cell. Fact.*	125	229	[5]
Lipases: sources, immobilization methods, and industrial applications	*Appl. Microbiol. Biotechnol.*	76	434	[1]
Driving Immobilized Lipases as Biocatalysts: 10 Years State of the Art and Future Prospects	*Ind. Eng. Chem. Res.*	54	718	[38]
Lipase immobilization with support materials, preparation techniques, and applications: Present and future aspects	*Int. J. Biol. Macromol.*	51	434	[46]
Recent advances in the improvement of enzyme thermostability by structure modification	*Crit. Rev. Biotechnol.*	50	99	[47]
Radio-frequency treatment for stabilization of wheat germ: Storage stability and physicochemical properties	*Innov. Food Sci. Emerg. Technol.*	50	3	[37]
Microbial lipases: An overview of screening, production and purification	*Biocatal. Agric. Biotechnol.*	46	409	[48]
Engineering *Yarrowia lipolytica* to Simultaneously Produce Lipase and Single Cell Protein from Agro-industrial Wastes for Feed	*Sci. Rep.*	43	109	[49]
Lipases in liquid formulation for biodiesel production: Current status and challenges	*Biotechnol. Appl. Biochem.*	40	220	[40]
A Versatile Approach for Enzyme Immobilization Using Chemically Modified 3D-Printed Scaffolds	*ACS Sustain. Chem. Eng.*	39	99	[50]
Current prospective in using cold-active enzymes as eco-friendly detergent additive	*Appl. Microbiol. Biotechnol.*	38	107	[51]
Make proper surfaces for immobilization of enzymes: Immobilization of lipase and alpha-amylase on modified Na-sepiolite	*Int. J. Biol. Macromol.*	37	774	[13]
A new heterofunctional support for enzyme immobilization: PEI functionalized Fe3O4 MNPs activated with divinyl sulfone. Application in the immobilization of lipase from *Thermomyces lanuginosus*	*Enzyme Microb. Technol.*	36	358	[52]
Novel lipases discovery specifically from marine organisms for industrial production and practical applications	*Process Biochem.*	36	280	[39]
Enzymes from Marine Polar Regions and Their Biotechnological Applications	*Mar. Drugs*	36	214	[53]
Solvent stable microbial lipases: current understanding and biotechnological applications	*Biotechnol. Lett.*	32	381	[14]
Remarkably enhanced activity and substrate affinity of lipase covalently bonded on zwitterionic polymer-grafted silica nanoparticles	*J. Colloid Interface Sci.*	32	204	[54]
Enhancing the Thermostability of *Rhizomucor miehei* Lipase with a Limited Screening Library by Rational-Design Point Mutations and Disulfide Bonds	*Appl. Environ. Microbiol.*	29	128	[55]
Biosensors and Bioassays Based on Lipases, Principles and Applications, a Review	*Molecules*	28	220	[56]
Evaluation of Strategies to Produce Highly Porous Cross-Linked Aggregates of Porcine Pancreas Lipase with Magnetic Properties	*Molecules*	27	640	[31]
Correlations of Molecular Weights of β-Glucans from Qingke (Tibetan Hulless Barley) to Their Multiple Bioactivities	*Molecules*	27	10	[57]
Substrate -Specificity of *Candida rugosa* Lipase and Its Industrial Application	*ACS Sustain. Chem. Eng.*	24	470	[33]
The Immobilization of Lipases on Porous Support by Adsorption and Hydrophobic Interaction Method	*Catalysts*	24	398	[35]
State-of-the-art strategies and applied perspectives of enzyme biocatalysis in food sector—current status and future trends	*Crit. Rev. Food Sci. Nutr.*	24	280	[58]
Enzymatic amidation for industrial applications	*Curr. Opin. Chem. Biol.*	24	56	[59]
Improved immobilization of lipase from *Thermomyces lanuginosus* on a new chitosan-based heterofunctional support: Mixed ion exchange plus hydrophobic interactions	*Int. J. Biol. Macromol.*	23	658	[60]
Biochemical aspects of lipase immobilization at polysaccharides for biotechnology	*Adv. Colloid Interface Sci.*	22	566	[6]
Production of Cold-Active Lipase by Free and Immobilized Marine *Bacillus cereus* HSS: Application in Wastewater Treatment	*Front. Microbiol.*	22	245	[34]
Recent Developments in Carriers and Non-Aqueous Solvents for Enzyme Immobilization	*Catalysts*	22	217	[17]
Immobilization of lipase B from *Candida antarctica* on epoxy-functionalized silica: characterization and improving biocatalytic parameters	*J. Chem. Technol. Biotechnol.*	22	155	[16]
Lipase immobilization on synthesized hyaluronic acid-coated magnetic nanoparticle-functionalized graphene oxide composites as new biocatalysts: Improved reusability, stability, and activity	*Int. J. Biol. Macromol.*	22	138	[15]
Validation of leaf enzymes in the detergent and textile industries: launching of a new platform technology	*Plant Biotechnol. J.*	22	39	[61]
Analysis of Aspergillus sp lipase immobilization for the application in organic synthesis	*Int. J. Biol. Macromol.*	21	696	[18]
Bioprocess development for L-asparaginase production by Streptomyces rochei, purification and in-vitro efficacy against various human carcinoma cell lines	*Sci. Rep.*	21	71	[62]
Synthesis and characterization of cross-linked enzyme aggregates (CLEAs) of thermostable xylanase from *Geobacillus thermodenitrificans* X1	*Process Biochem.*	20	226	[63]
Enzyme-inorganic hybrid nanoflowers: Classification, synthesis, functionalization and potential applications	*Chem. Eng. J.*	20	187	[64]
Feruloyl esterase immobilization in mesoporous silica particles and characterization in hydrolysis and transesterification	*BMC Boichem.*	20	204	[65]

## 4. Network Visualization of Keyword Co-Occurrence

Keyword co-occurrence analysis assumes that words often appear together and have a thematic relationship [22]. VOSviewer works with aggregated relationships and shows the strong relationship between the items. Thus, distance-based maps are generated, where a smaller distance indicates a stronger relationship between terms. The lines drawn between the items indicate the relationships, and the items with greater prominence in the graph are considered of greater importance within the investigated context [21].

**Figure 3:**
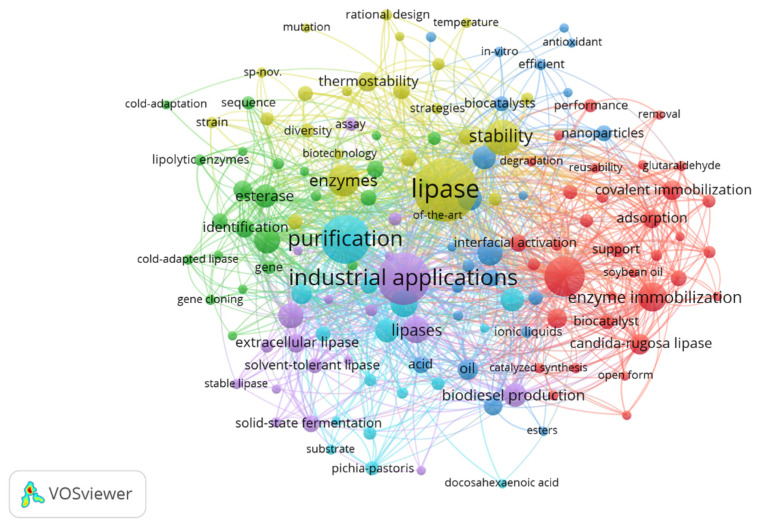
Network visualization map of the co-occurrence of keywords based on the number of publications, considering the period from 2018 to 2022. The distance between terms shows the relationship’s strength (link strength), and the colors indicate a set of keywords with a high degree of simultaneous occurrence (cluster). The items that are more prominent are more frequent in database searches.

In order to identify possible topics researched in the last five years, a network was built for the co-occurrence of keywords. From the information extracted from the WS, the minimum number of five occurrences for a keyword was established, resulting in a total of 140 keywords identified for constructing the network graph in VOSviewer, as presented in Figure 3. When the item relationships were analyzed through the drawn lines, it was possible to observe the formation of six clusters, highlighted by the colors in the visualization map.

With the grouping of terms, it was possible to identify the existing relationships among topics in the field of research of enzyme immobilization, studies on enzymatic catalysis, its characteristics, and studies on enzymes from microbial sources (cluster 1, in red). In cluster 2 (in green), the search terms were enzymes, cold-adapted lipases, enzyme identification, and classification. Cluster 3 (dark blue) presented a search set involving nanoparticle stability, in vitro antioxidant stability, transesterification, esterification, and biodiesel. The terms in cluster 4 (in yellow) are related to research on biotechnology, directed evolution of enzymes, stability, and enzyme stability. In cluster 5 (in purple), the researched topics were directed to the production and application of lipases, in which the terms industrial application, biochemical characterization, extracellular lipase, production of lipases, solid-state fermentation, and microbial lipases appear. Cluster 6 (in light blue) contains search terms related to enzyme production, optimization, hydrolysis, fermentation, substrate, purification, alkaline lipases, and microbial lipases.

Co-occurrence links present the strength of the relationship between items (link strength), represented by a positive numerical value. Table 2 presents keywords with a value ≥112 total link strength, identified in VOSviewer. The higher this value, the stronger the link, indicating the number of publications in which two terms occur together [33]. The keywords lipase, industrial applications, purification, immobilization, stability, and enzymes were the most frequent shared search terms from 2018 to 2022, evidencing possible most sought-after research topics related to lipases.

## 5. Journal Co-Citation Map

**Figure 4:**
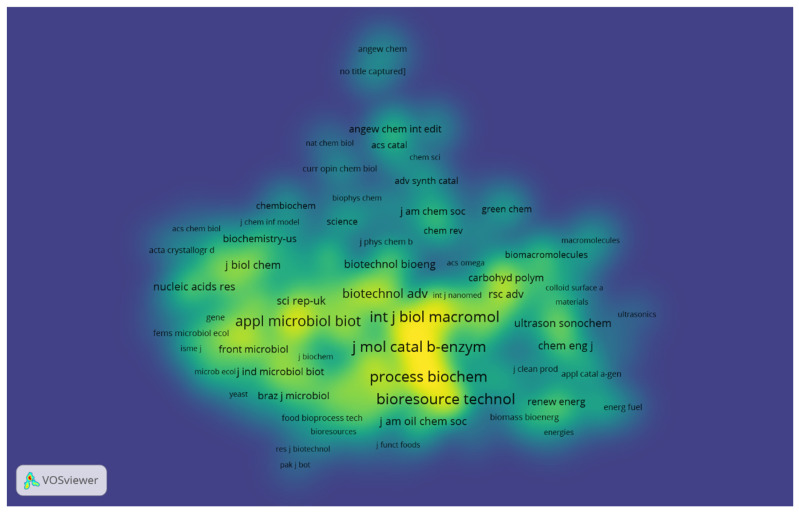
Density visualization map for co-citation of journals with at least ten citations. The most prominent areas show the journals that received the most citations.

The journal co-citation map shows the structure of the researched scientific areas. For the construction of the map, journals with a minimum of ten citations were selected, totaling 429 journals used to construct the co-citation map. Figure 4 shows the density view, where each point on the map is represented by a color related to the journals’ density at that point. Thus, it is possible to observe that the following journals: *Journal of Molecular Catalysis B: Enzymatic*; *International Journal of Biological Macromolecules*; *Bioresource Technology*; *Process Biochemistry*; *Enzyme and Microbial Technology*; *Applied Microbiology and Biotechnology*; *Biotechnology and Applied Biochemistry*; and *Biotechnology Advances* have very dense and highlighted areas on the map, showing a more significant number of citations in these journals in the investigated period. In addition, it is possible to observe on the map that these journals are located close to each other, indicating closely related research fields. 

Analyzing the scientific areas covered by the journals, it is possible to perceive the connection among them. These journals generally focus on investigating applications of enzymes; the chemical and biological aspects of macromolecules; studies on bioprocesses; processes involving the use of enzymes, microorganisms, animal cells, and plant cells; and advances in the areas of biochemistry and biotechnology. Gonçalves et al. [24] indicated the journals *Journal of Molecular Catalysis B: Enzymatic*; *Process Biochemistry*, and *Enzyme* and *Microbial Technology* hold great relevance for research mainly related to enzyme immobilization. Therefore, it was possible to obtain an overview of the structure of the co-citation map of the journals, to observe the intellectual links among journals, and the impact of this network through the visualization of the density map (Figure 4).

## 6. Main Challenges Reported on Lipase Application

Lipases have essential applications in promoting various biochemical processes in the industry, with an increasing demand for production, for example, for fresh beef monitoring [66], biodiesel synthesis [67,68,69,70], and immobilization [71,72,73]. The selectivity of lipases contributes to the synthesis of food products that are beneficial to human health, such as oil supplements, omega 3, and omega 6, for example. They also contribute to the decomposition of complex fatty acids and modification of egg proteins, production of emulsifiers, shelf life extension and texture improvement in bakery products, hydrolysis of milk fats and ripening of cheeses in dairy industries, and flavor production [1,5]. In this scenario, lipases of microbial origin have been gaining prominence. Much research has been conducted on isolating, screening, and critical growth parameters for microbial strains to achieve the maximum yield in the production of these enzymes [33,48].

Enzyme-catalyzed reactions have been increasingly applied in the most diverse industries. In general, the enzymatic catalysis processes are characterized by milder conditions, high selectivity, lower energy consumption, and reduced waste production. Nevertheless, stabilizing these biocatalysts is still challenging, which reflects limitations in the large-scale implementation of enzymatic processes [30,32].

To overcome these limitations, studies to understand the dynamic behavior of biochemical reactions over time, described by kinetic data, are indispensable. This information is vital for computational modeling, creating network models of biochemical reactions, and helping to better understand living cell processes. However, developing kinetic models requires this information to be integrated and ordered; this highlights the importance of the development of databases, for example of enzymes and proteins, that can store kinetic parameters to aid in the development of computational modeling and scale-up studies [74,75].

The modeling of biotransformations is fundamental to the design of enzyme-catalyzed reactions. Understanding enzyme kinetics allows the determination of optimal operational parameters and the identification of the most efficient production system since the determination of reaction kinetic parameters is fundamental for equipment design both in the laboratory and in industrial production. Most enzymatic reactions occur under mild reaction conditions and the concentration of substrate and product has the greatest influence on the determination of enzyme kinetics. Therefore, it is important to know the mechanism behind the interactions between enzyme and substrate, and enzyme and product. In addition, precise measurements of substrate concentration and reaction time are essential to accurately determine kinetic parameters [75,76].

Studies are needed to correlate the knowledge of genetic and computational tools to improve the properties of lipases, such as increased activity, productivity, thermal stability, reuse, and enantioselectivity, as well as meeting industrial demands with increasingly sustainable processes [32,58]. Li et al. [55] developed studies with lipases from *Rhizomucor miehei*, applying various computational design methods. The authors showed that point mutations and engineered disulfide bonds could reduce the number of clones selected to increase the strain’s thermal stability. Xu et al. [47], in a literature review, showed that computational studies aimed at developing protein modifications are of fundamental importance for the production of enzymes with better performance, for instance, thermostable enzymes for industrial applications.

## 7. The Impact of Enzyme Immobilization on Industrial Processes

In general, soluble enzymes do not present characteristics that allow their application in large-scale continuous processes. With their sensitivity to process conditions, low stability, and difficult reuse at an industrial scale, studies focus on immobilization techniques to improve this performance [77]. When we talk about lipases, we have as an exception Eversa^®^ Transform 2.0 (Eversa), which is one of the few liquid lipase formulations for industrial application. A genetically-modified lipase, derived from the lipase of the fungus *Thermomyces lanuginosus* [77,78], with low cost, can be used in processes without necessarily being reused [79,80]. Currently, its major focus of application and studies covers the biofuel industry; however, it still presents a broad potential for evaluation in other sectors [81,82,83].

The main goal of immobilization techniques is the protection of enzymes under extreme environmental conditions, including the influence of temperature, pH, and organic solvents during the application processes, allowing enzymatic activity to be maintained even under various reaction conditions since the main function of the support is to stabilize the enzyme structures and, consequently, to preserve enzyme efficacy and storage. Immobilized enzymes are more robust, show higher stability, and are easier to handle compared to their soluble forms. They are also easily recovered/recycled after use [78,84,85,86,87,88].

Enzyme immobilization methods include physical adsorption, ionic and covalent bonds, and various techniques such as binding, entrapment, encapsulation, and reticulation. Various organic and inorganic materials or carriers can be used to apply enzyme immobilization techniques [88,89]. Immobilization has been observed as a promising and effective technology to expand the applicability of enzymes in industrial environments, promoting easier downstream processing and greater process control in different reactor configurations, such as packed bed reactors, fluidized beds, and tank reactors [31,90].

In the food industry, for example, studies show that immobilized enzymes are applied in different stages of food and beverage processing, in brewing, meat tenderization, baking, and protein hydrolysis, offering numerous advantages such as stabilization and reuse, which enable cost reduction [77,78,91,92]. They are also used in several enzymatic biosensors for the analysis of food quality [79,80,93,94].

Lipase immobilization techniques have been the subject of several studies developed over the years [6,13,16,81,82,83,84,95,96,97,98]. These techniques improve recyclability, stability, and enzymatic activity, impacting market productivity and profitability. This was demonstrated in the review research performed by Chandra and collaborators [5]. The authors emphasize that it is essential to choose the immobilization technique and the type of carrier, which is strongly influenced by efficiency and its biocompatibility, chemical and thermal stability, insolubility, and reuse. In other words, knowledge of protein engineering, enzyme immobilization, process engineering, and life cycle analysis is fundamental to establishing a method compatible with the enzyme under study. This highlights the relevance of research involving enzyme immobilization [15,50,65], which directly impacts the optimization of bioprocesses.

## 8. Scaling Up the Lipase-Catalyzed Process

**Figure 5:**
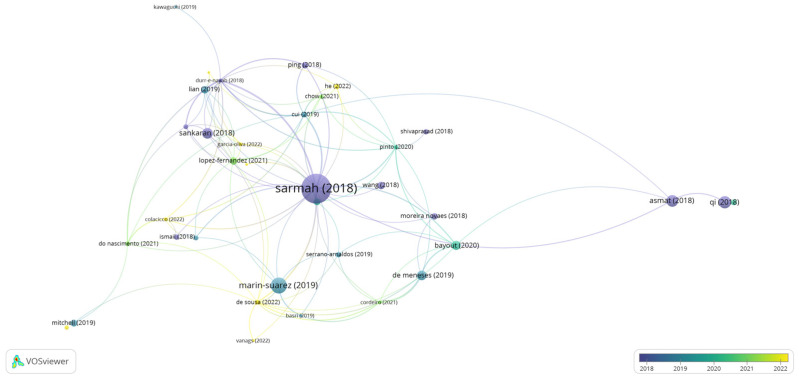
Network visualization map for the co-occurrence of works that bring information about the scale-up process using lipases applied to bioprocesses [41,99,100,101,102,103,104,105,106,107,108,109,110,111,112,113,114]. Publications in the period from 2018 to 2022 were considered. The distance between the terms shows the strength of the relationship (link strength), and the colors indicate a set of keywords with a high degree of simultaneous occurrence (cluster). More prominent items are more frequent in database searches.

In the graph generated by the program VOSviewer (Figure 5), it is observed that the article by [1] presented the highest number of citations and links, regarding the topic of scaling-up for catalytic processes employing lipase. In this specific work by [1], studies and advances are compiled within the industrial application of the enzyme, in which it cites some properties of lipase that are a focus on improvement for scale-up such as activity, productivity, thermostability, reuse, enantioselectivity, and tolerance to organic solvents, which is also cited in the works of [6,13]. Concerning the process, the need surrounds the optimization of the design and operating parameters of the plants, as well as mapping the economic viability of processes catalyzed by lipases [1,14,15].

Going deeper into these details, the authors address the relevance of recombinant technology studies with protein engineering, which act in the structural modification of this enzyme [16,18], mainly of the active site, and in the expression of this biocatalyst in a fungal matrix [17,21,22]. Another factor explored by [1,23] for scale-up is immobilization, which was detailed in the previous topic and can also be observed in the works of [14,27,29], being [16,30] grouped by similarity in citations number, however, with few links with the other articles present in Figure 5.

Modeling and optimization by statistical experimental planning stand out in most of the works with lipase [1,31]. Nonetheless, optimization alternatives such as a neural network-based genetic algorithm have also been explored in a complementary way, as well as hydrodynamic modeling [36] and kinetic studies [32,38]. Some authors, such as [1,21,24,28,32,36], have directed their studies to the bioreactor part and the limitations that may occur during the process, as well as identifying the optimizations to be made. The author of [28] focused his studies on mass transfer, using a fixed bed reactor (FBR) with immobilized lipase, in which he observed that external mass transfer limitations affect the overall reaction rate for palm olein hydrolysis. The authors of [1] researched scale-up for stirred tank reactors (STR), where different approaches were required for energy consumption and cost-effectiveness. Since there were limitations in enzyme-substrate interaction due to the accumulation of the immobilized enzyme at the bottom of the reactor, [38] used the spinning mesh disc reactor (SMDR), conciliating a recent technology with a low-cost immobilized lipase, and obtained effective results for scale-up compared to the batch reactor, indicating that future work addressing the optimization of reaction conversion by a change in the support and catalyst for better compatibility with solvents under study. 

Among the observed works, when talking about the bioreactor, there is a range of studies that need to be considered for the effective dynamics of the reaction system, which should be considered for scale-up. Thus, the large amount of biocatalyst is mentioned, in relation to the efficiency of the reaction and the enzymatic activity, as well as the impregnation of the material on the reactor wall in continuous processes or for long periods, the adequate heat and mass transfer, the effective operational control, the combination of the reaction medium with the characteristic of the enzyme, the kinetic model of the reaction, and the amount of water in the medium [21,32,47].

## 9. Conclusions

Lipases stand out in different industrial sectors with wide applicability; consequently, the number of research works involving this enzyme covers multiple areas of knowledge. Therefore, the systematic review presented aimed to map the potentiality of this biocatalyst aiming at its industrial application, where the following topics can be highlighted:

- The works with significant numbers of citations found in the search focus on lipase immobilization processes for application in industrial processes.
- Within this niche, the application is focused on the biotechnology and bioprocess sectors, which include food, cosmetics, biofuels, and environmental areas, which show the versatility of this enzyme.
- When observing the topics that comprise the optimization of lipases, studies are found evaluating both the supports for immobilization, fermentative processes of enzyme production, enzymatic purification, biochemical characterization, and the application in reactions in both an organic and inorganic media, reinforcing the breadth of use of this biocatalyst.
- It is verified that in the case of lipase immobilization techniques, the tests comprise the configuration of support materials, operational stability, and reuse.
- Kinetic studies are fundamental in this process of transition from laboratory scale to industrial scale. Allied with the computational modeling of these reactions, it allows us to determine and optimize operational parameters, in addition to enabling monitoring of the bioprocess and planning the structure necessary for implementation.
- Other factors that contribute to the previous topic are the genetic studies at the structural level of lipases, which allows the improvement of their characteristics regarding activity, productivity, thermal stability, reuse, and enantioselectivity.
- Compatibility studies considering the synergy of free or immobilized enzyme, reactor type, reaction medium, and product are fundamental to performing a combination that allows an efficient process, with high yields, and continuous operation processes.

Thus, the approach of enzyme technology focused on lipases is broad, requiring the combination of knowledge in protein engineering, immobilization, process engineering, and life cycle analysis to both understand the mechanisms that involve this biocatalyst and enable the use on an industrial scale with the least efficiency loss in the processes.

## Figures and Tables

**Table 2:** Number of occurrences of terms with value ≥ 112 total link strength.

Keyword	Total Link Strength	Occurrences
Lipase	784	147
Industrial applications	559	103
Purification	523	91
Immobilization	389	65
Stability	310	53
Enzymes	231	43
Enzyme immobilization	209	37
Optimization	191	34
Lipases	168	33
Expression	190	32
Biochemical characterization	181	29
Biodiesel	176	29
Biocatalysis	163	28
Biodiesel production	160	26
Esterase	146	26
Hydrolysis	158	26
Enzyme	164	25
Microbial lipases	146	25
Alkaline lipase	145	21
Candida rugosa lipase	127	21
Oil	126	20
Covalent immobilization	124	19
Kinetic resolution	123	19
Thermostability	123	19
Cloning	112	18
Extracellular lipase	128	18
Identification	118	18
Adsorption	112	17

## Data Availability

The data presented in this study are available on request from the corresponding author.
